# Primed to be inflexible: the influence of set size on cognitive flexibility during childhood

**DOI:** 10.3389/fpsyg.2014.00101

**Published:** 2014-02-12

**Authors:** Lily FitzGibbon, Lucy Cragg, Daniel J. Carroll

**Affiliations:** ^1^Department of Psychology, University of SheffieldSheffield, UK; ^2^School of Psychology, University of NottinghamNottingham, UK

**Keywords:** cognitive flexibility, development, priming, executive function, set size, rule representation

## Abstract

One of the hallmarks of human cognition is cognitive flexibility, the ability to adapt thoughts and behaviors according to changing task demands. Previous research has suggested that the number of different exemplars that must be processed within a task (the set size) can influence an individual's ability to switch flexibly between different tasks. This paper provides evidence that when tasks have a small set size, children's cognitive flexibility is impaired compared to when tasks have a large set size. This paper also offers insights into the mechanism by which this effect comes about. Understanding how set size interacts with task-switching informs the debate regarding the relative contributions of bottom-up priming and top-down control processes in the development of cognitive flexibility. We tested two accounts for the relationship between set size and cognitive flexibility: the (bottom-up) Stimulus-Task Priming account and the (top-down) Rule Representation account. Our findings offered support for the Stimulus-Task Priming account, but not for the Rule Representation account. They suggest that children are susceptible to bottom-up priming caused by stimulus repetition, and that this priming can impair their ability to switch between tasks. These findings make important theoretical and practical contributions to the executive function literature: theoretically, they show that the basic features of a task exert a significant influence on children's ability to flexibly shift between tasks through bottom-up priming effects. Practically, they suggest that children's cognitive flexibility may have been underestimated relative to adults', as paradigms used with children typically have a smaller set size than those used with adults. These findings also have applications in education, where they have the potential to inform teaching in key areas where cognitive flexibility is required, such as mathematics and literacy.

## Introduction

One of the hallmarks of human cognition is its flexibility. People are capable of flexibly adapting their thoughts and behaviors according to novel or changing environmental demands or task goals. For example, when switching between a Mac and a PC, different responses are often required to achieve the same goal, such as pressing a button in the top-left or top-right corner to close a browser window. Cognitive flexibility in adults and children is affected by the set size of the tasks involved—that is, the size of the pool of different stimuli that must be processed in the task (Kray and Eppinger, 2006; Kray et al., 2012). When a large pool of stimuli are used (a large set size), performance is better than when a small pool of stimuli are used (a small set size).

Set size is of theoretical importance because it informs the debate regarding the roles of top-down cognitive control and bottom-up priming in the development of cognitive flexibility (Cepeda et al., 2001). Set size is also methodologically important because one of the crucial differences between cognitive flexibility paradigms used with adults and those used with children is their set size (Cragg and Chevalier, 2012). Cognitive flexibility paradigms used with young children typically use a small number of stimuli (e.g., the Dimensional Change Card Sort, DCCS, Zelazo, 2006; and Shape School, Espy, 1997). In contrast, paradigms used with adults typically use a much larger set size (Rogers and Monsell, 1995; Richter and Yeung, 2012). Understanding the influence of set size in cognitive flexibility development can also better inform early school education for children in key areas such as mathematics and literacy where cognitive flexibility plays a central role (Bull and Scerif, 2001; St Clair-Thompson and Gathercole, 2006; Blair and Razza, 2007; Bull et al., 2008; Yeniad et al., 2013). This paper explores what role set size plays in cognitive flexibility during the early school years. We will first describe the development of cognitive flexibility during the early school years, and then discuss possible explanations for the role that set size might play in children's ability to switch flexibly between tasks.

When studying children's cognitive flexibility, researchers typically use paradigms that involve switching between two simple tasks, such as matching stimuli by their color and matching stimuli by their shape (Zelazo, 2006). By 3 years, children can perform either task well on its own, but typically fail to switch from one to the other (Zelazo et al., 1996). By 4 years, children can reliably make a single switch from one task to another (Zelazo et al., 1996) but experience difficulty switching flexibly back and forth between the two tasks (Carlson, 2005; Hongwanishkul et al., 2005; Moriguchi and Hiraki, 2013). Around the age of five, children become able to flexibly switch back and forth between simple tasks (Chevalier and Blaye, 2009). At this age, response time starts to be a reliable metric of children's cognitive flexibility. This allows nuanced questions about the component processes required for cognitive flexibility to be investigated (Best and Miller, 2010). From around this age children are more likely to respond more slowly and less accurately when asked to switch from one task to another (i.e., on switch trials) than when asked to repeat the same task (i.e., on non-switch trials) (Dauvier et al., 2012). This is known as the switch cost. Switch costs tend to decrease with age (Crone et al., 2006; Huizinga et al., 2006; Chevalier and Blaye, 2009; Cragg and Nation, 2009; however this developmental trend is not reliable after the preschool years: see Dibbets and Jolles, 2006; Karbach and Kray, 2007; Kray et al., 2012). Switch costs do not diminish completely. They can reliably be found when adults must switch between tasks (for a review see Kiesel et al., 2010).

Examining switch costs in young children allows us to address important questions, such as what processes contribute to switch costs, and how these processes change during development (Best and Miller, 2010). Lessons drawn from adult participants suggest that switch costs reflect two distinct types of cognitive process. First, top-down control processes contribute to switch costs. These include the retrieval of task rules and the deliberate shifting of attention toward task-relevant stimulus attributes which are required on switch trials, but not on non-switch trials (Rogers and Monsell, 1995; Meiran, 1996; Monsell, 2003). Second, bottom-up priming processes contribute to switch costs. These include the priming of associations between stimuli and responses that build up over successive trials and facilitate non-switch trials but are detrimental to switch trials (Allport and Wylie, 2000; Waszak et al., 2003). It is not yet well understood to what extent each of these processes contributes to switch costs in children (see Cragg and Chevalier, 2012 for a review).

In this paper we explore the role of set size in the development of cognitive flexibility in children aged between 4 and 12 years. In particular, through manipulations of set size, we investigate the relative roles of top-down rule representation and bottom-up stimulus-task priming on cognitive flexibility during the early school years. The following sections explain how rule representation and stimulus-task priming relate to set size and the development of cognitive flexibility.

One mechanism by which set size would likely affect cognitive flexibility is through the way that task rules are represented. Consider a paradigm where children must switch between matching stimuli by their colors and matching them by their shapes. With a small set size [when the only colors in the task are (blue) and (red)], task rules can be efficiently represented in stimulus-specific terms—for example, “red blocks go in the red box” and “blue blocks go in the blue box.” However, with a large set size, (for example, when there are many colors), it would be highly inefficient to formulate one rule for each individual color. It would be far more efficient to represent the task rules in abstract, dimension-level terms, such as “put the blocks into boxes that are the same color.” Indeed, large pools of stimuli have been found to promote abstract categorization in toddlers (Perry et al., 2010). It is thus plausible that large and small set sizes create quite different task representations: a large set size on a task is likely to engender more abstract, dimension-level representations of task rules, whereas a small set size may engender more stimulus-specific representations of task rules.

Relevant to the relationship between set size and cognitive flexibility, evidence suggests that the way rules are represented determines how flexibly they can be switched between. It has been suggested that the early development of the prefrontal cortex supports abstract representation of task rules (Munakata et al., 2011) and cognitive flexibility (Moriguchi and Hiraki, 2013). Changes in the way that task rules are represented, from stimulus-specific representation to dimension-level representation, leads to better cognitive flexibility performance (Kharitonova et al., 2009; Kharitonova and Munakata, 2011). Thus, it is plausible that a large set size facilitates cognitive flexibility, by engendering dimension-level representation of task rules, and that smaller set sizes hinder rule switching, by engendering stimulus-specific rule representation.

The second mechanism by which set size would likely affect cognitive flexibility is through stimulus-task priming. Stimulus-task priming refers to the bottom-up process by which prior experience on a task leads to pairings that have been previously experienced being preferentially activated on later trials, regardless of whether they are currently task-relevant or not (Reuss et al., 2011). When the set size is small, individual stimuli appear more frequently—so there is more stimulus repetition—than when the set size is large. Associations between specific stimuli and specific tasks are thus more likely to build up with small set sizes than with large set sizes. Stimulus repetition has been shown to cause stimulus-task priming in adults, which contributes to greater switch costs (Waszak et al., 2003; Koch and Allport, 2006). Stimulus repetition is also detrimental to cognitive flexibility in preschool children (Müller et al., 2006; Experiment 3).

There are two reasons for thinking that stimulus-task priming might inflate switch costs. Firstly, on non-switch trials where the task repeats, if the stimulus was already associated with that task, then responses should be faster and more accurate because the currently relevant task was primed by the stimulus. Secondly, on switch trials, if the stimulus was associated with the previous (but no longer relevant) task, then responses should be slower and less accurate because the incorrect task was primed by the stimulus (Waszak and Hommel, 2007). Indeed, in a voluntary switching paradigm, where participants could choose to respond with a task repetition or a task switch on each trial, stimulus repetition was found to bias a task repetition response and stimulus change was found to bias a task switch response (Mayr and Bell, 2006).

To our knowledge only two studies have directly explored the effects of set size on cognitive flexibility in a developmental context (Kray and Eppinger, 2006; Kray et al., 2012). The first of these studies compared cognitive flexibility in young (*M* = 21 years) and older adults (*M* = 66 years) on a large and a small set size (Kray and Eppinger, 2006). It was found that the small set size induced greater switch costs than the large set size, but this effect was only seen in the older adults.

The second study (Kray et al., 2012) assessed cognitive flexibility in two groups of children (4- to 6-year-olds and 7- to 10-year-olds) and one group of young adults. Set size affected cognitive flexibility in two ways. First, when the set size was large, older children were better able to ignore task-irrelevant information than when set size was small. This effect was not seen for younger children or adults. Second, there was an effect of set size on conflict adaptation in older children. In some trials, both tasks would lead to the same response (compatible trials) so there was no conflict. In other trials, the relevant task would lead to one response and the non-relevant task would lead to the other response (incompatible trials) so there was conflict between the two tasks. The older children made greater control adjustments following incompatible trials in the small set size condition than the large set size condition. This suggests that the conflict that occurs between tasks is more salient with small set sizes than with large set sizes, and consequently results in better adjustment of control processes following its occurrence.

The absence of set size effects in younger children was surprising given the known developmental trends described above in both abstract rule representation (Munakata et al., 2012) and stimulus-task priming (Hommel et al., 2011). Furthermore, there is indirect evidence to suggest that preschool children's cognitive flexibility may be enhanced by increasing the task set size. For example, preschool children's ability to switch tasks has been improved by increasing the set size on the DCCS from two colors and two shapes to four colors and four shapes (Fisher, 2011a). However, the number of response options was also increased from two to four in that experiment, so it is unclear which of the two methodological changes was responsible for the facilitative effect. The absence of a set size effect in the youngest children in Kray et al.'s (2012) study may have been due to what the authors describe as the high “general demands on cognitive control processing” that the experimental paradigm entailed (Kray et al., 2012, p. 127). These high demands and the length of the test period may have also resulted in a high exclusion rate for the youngest age group (35%). Clearly, set size influences cognitive flexibility. However, the somewhat ambiguous findings indicate that further investigation is necessary.

Perhaps the most surprising finding was that increasing the set size did not reduce children's switching costs. This stands counter to the predictions drawn from the broader literature, and indeed counter to Kray et al.'s (2012) own predictions. However, this surprising absence of set size effect may in part have been due to the paradigm used. In one task, children categorized pictures as “animals” or “objects.” This differs from typical developmental cognitive flexibility paradigms, which tend to be based on perceptual rather than conceptual features of the stimuli (FIST, Jacques and Zelazo, 2001; DCCS, Zelazo, 2006). Children are typically required to judge the color or shape of a stimulus, rather than its conceptual category membership. Conceptual categorization is a perfectly legitimate construct to study, though its use in cognitive flexibility paradigms may have attenuated the effects of set size in Kray et al.'s (2012) study for two reasons. First, children's perceptual processing is more robust than their conceptual processing (Fisher, 2011b), which would likely lead to stronger stimulus-task priming for perceptual than conceptual features. Second, there is also evidence to suggest that priming can occur at the level of semantic category as well as for individual stimuli (Waszak et al., 2004). Semantic category-level priming may have attenuated the facilitative effects of a large set size on switching costs.

The two experiments in this paper use the Switching Inhibition and Flexibility Task (SwIFT, Carroll and Cragg, 2012). This is a developmentally appropriate measure of cognitive flexibility that requires children to match stimuli according to their color or their shape. This kind of perceptual processing is known to be robust in young children (Zelazo et al., 1996; Fisher, 2011b). Processing demands that are orthogonal to cognitive flexibility are minimized: the goal setting demands are minimal because the task is cued on every trial with a transparent auditory cue (the word “color” or “shape”—Chevalier and Blaye, 2009). The working memory demands are low because the responses are intuitive so there is no need to maintain the appropriate responses for each task. The SwIFT thus gives a relatively pure measure of the costs of switching from one task to another by eliminating orthogonal processing costs that are present in other cognitive flexibility paradigms.

A review of the cognitive flexibility literature in general would lead us to predict a clear set-size effect on switch costs, although the few direct tests are tantalizingly inconclusive. On the basis of previous findings we would predict that a large set size would lead to smaller switch costs, and that a small set size would lead to larger switch costs. We expected that this effect would be largest in the youngest children, and would diminish with age. There were two reasons for this prediction: first, young children are less likely to spontaneously represent task rules in abstract, dimension level terms than older children (Kharitonova et al., 2009) and so would benefit most from a manipulation that engendered this type of rule representation. Second, children are more susceptible to stimulus-task priming than adults (Hommel et al., 2011), and if this relationship is linear, then younger children would be expected to be more affected by stimulus repetition than older children. We also expect to see a reduction in switch costs more generally during the early school years in line with findings from the Advanced DCCS paradigm (Chevalier and Blaye, 2009).

## Experiment 1

### Methods

#### Participants

One hundred and forty nine 4- to 11-year-old children (80 female) were randomly selected from a larger sample attending Summer Scientist Week, a science engagement event at the University of Nottingham. Children were randomly allocated to one of two conditions: large set size or small set size. Each condition was further subdivided by age to give three similarly sized groups: 49 in the youngest age group (4;0- to 6;6-year-olds, *M* = 5;2, *SD* = 0;8, 27 females); 50 in the middle age group (6;7- to 8;4-year-olds, *M* = 7;4, *SD* = 0;6, 24 females); and 50 in the oldest age group (8;5- to 11;9-year-olds, *M* = 9;10, *SD* = 1;0, 29 females). Six further children were excluded because of missing data. Participants had no reported developmental disorders or special educational needs. Children's standardized scores on the British Picture Vocabulary Scale (BPVS) did not differ between the two test conditions [small set size: *M* = 109.29, large set size: *M* = 109.73, *t*_(138)_ = −0.18, *p* = 0.86]. This was indicative of similar levels of general cognitive functioning in the two groups. BPVS scores were missing from nine participants. All the children were tested individually in a quiet room. Parental consent for participation in this research was obtained for all participants. The experimental procedure was approved by the School of Psychology Ethics Committee at the University of Nottingham.

#### Materials

Stimuli were presented and responses recorded on an Iiyama ProLite touch screen monitor connected to a Samsung P510 PC laptop running PsychoPy software (Peirce, 2007). Children responded by touching the relevant part of the screen and the program recorded their responses. The stimuli used in this task were nine simple novel shape outlines filled in with solid colors. The nine colors were all of equal saturation and brightness, and their hues were evenly distributed on a color wheel. Each image was approximately 6 × 8 cm.

#### Procedure

Children played a simple matching game. They were shown a prompt stimulus on a touchscreen computer, followed by two response stimuli. The prompt stimulus always had two dimensions (color and shape). On each trial, one response stimulus matched the prompt's color, and the other response stimulus matched the prompt's shape. Children were told to select the response stimulus that matched the prompt on the dimension relevant to the task for that trial (always either color or shape).

Trials were presented in two phases: the rule-learning phase and the task-switching phase. In the rule-learning phase there were two pure blocks of trials (6 trials each), in which children performed the same task for every trial. In one pure block children were required to match the stimuli by color, and in the other pure block they were required to match the stimuli by shape. The order of the pure blocks was counterbalanced between participants. In the task-switching phase there were three mixed blocks. During the mixed blocks (12 trials each) children were required to switch between the two tasks. The order of trials was pseudo-randomized such that some trials required children to perform the same task as the trial before (non-switch trials) and others required children to perform a different task to the trial before (switch trials). The first trial of each block was neither a non-switch nor a switch trial. There were a total of 16 non-switch trials and 17 switch trials. The number of trials was chosen to be developmentally appropriate for the younger participants, and was in line with previous research with 4- to 6-year-old children on the Shape School and Advanced DCCS paradigms (Zelazo, 2006; Chevalier et al., 2010; Blaye and Chevalier, 2011).

Before each pure block, children were shown an example trial using the standard task array of a prompt and two response stimuli. The task rules and the correct way to respond (touching the appropriate response stimulus with the index finger) were explained by the experimenter. The first pure block was also preceded by two practice trials. Feedback for correct and incorrect performance was given after each practice trial in the form of on-screen text and verbal feedback from the experimenter. For all experimental trials no feedback was given. If any practice trials were completed incorrectly two further practice trials were presented.

**Figure 1:**
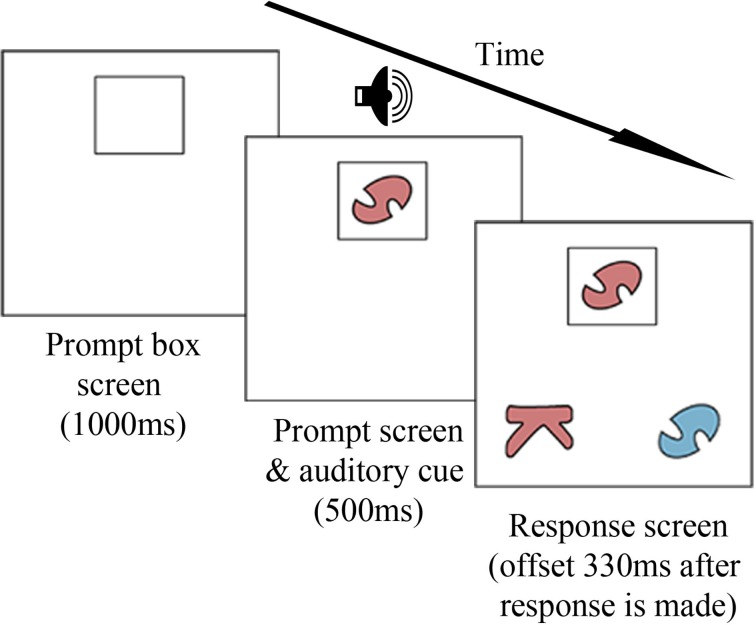
**Trial procedure**. The prompt stimulus (**top**) matches one response stimulus (bottom **left**) according to its color and the other response stimulus (bottom **right**) according to its shape. An auditory cue (“color” or “shape”) is onset concurrently with the prompt stimulus.

A graphical representation of the trial procedure can be found in Figure 1. Each trial began with a white screen showing a black outline of a rectangle (the prompt box) located at the top center of the screen. After a delay of 1000 ms, the prompt stimulus appeared in the prompt box, together with an auditory cue (a female voice saying “color” or “shape,” as appropriate for that trial). After a further delay of 500 ms, two response stimuli appeared in the bottom left and right corners of the screen. One response stimulus matched the prompt on the color dimension, and the other response stimulus matched the prompt on the shape dimension. Neither response stimulus ever matched the prompt stimulus on both dimensions. All stimuli remained on the screen until children responded. Testing lasted approximately 15 min.

The experiment used a between-participants design, and there were two conditions, differing only in terms of set size. In the small set size condition there were two exemplars of color and two exemplars of shape (meaning that there were four stimuli in total). As in the Advanced DCCS, the target stimuli were kept constant, although their positions were counterbalanced. Within each block, each prompt stimulus was displayed six times, an equal number of times on color and shape trials, and approximately an equal number of times on non-switch and switch trials. There was an average of one intervening trial between recurrences of the same prompt stimulus.

In the large set size condition there were nine exemplars of color and nine exemplars of shape (meaning that there were 81 bidimensional stimuli in total). Stimulus selection was constrained so that a prompt stimulus never appeared more than once within a block, and the color and shape values never repeated on consecutive trials. On approximately half of the trials, one of the dimension values (the color or the shape) had occurred previously within the block. There were on average four intervening trials between recurrences of a dimension value within each block.

### Results

All analyses were performed after excluding the first trial from each block, since these trials were neither switch nor non-switch trials. Trials with RTs less than 200 ms or greater than 10,000 ms, and trials that were 2.5 standard deviations away from the individual's mean RT for that type of trial (5.4%), were excluded from the response time analysis. The response time analysis also included only correct trials that also followed a correct trial. This is because only these trials can be definitively classified as a non-switch trial or a switch trial. The mean number of trials entered into the analysis did not differ between the set size conditions, nor between switch and non-switch trials. Younger children contributed fewer trials to the analysis than older children because of higher error rates (*M*s = 23.4, 25.4, and 27.4 trials entered for the youngest, middle and oldest age groups respectively). A natural logarithmic transformation was applied to the response time data to control for baseline changes in response time with age (Meiran, 1996; Chevalier et al., 2010). For clarity, untransformed RTs are presented in figures and tables.

Analyses were conducted separately for each of two dependent variables: mean accuracy and mean log-transformed RTs. To assess switch costs, mixed-measures ANOVAs were performed with trial type (non-switch vs. switch) as a within-participants variable, and age group (youngest vs. middle vs. oldest) and set size (small vs. large) as between-participants variables. We chose to use trial type (non-switch and switch) as a within-subjects variable rather than the difference score because this controls for both overall performance and processing speed differences between the experimental groups.

The analysis of accuracy data revealed a main effect of age, *F*_(2, 143)_ = 10.24, *p* < 0.001, η^2^ = 0.13. Bonferroni-adjusted *post-hoc* tests revealed that the youngest group was less accurate than both the middle and oldest age groups (*p*s < 0.05). The middle age group was also less accurate than the oldest age group (*p* < 0.05). Analysis of trial type revealed a significant switch cost, *F*_(1, 143)_ = 50.47, *p* < 0.001, η^2^ = 0.26, with less accurate performance on switch trials than non-switch trials (see Table 1 for a summary of the means). Accuracy switch costs were not significantly different between age groups. There was no effect of set size on the accuracy of performance. This indicates that there were no baseline differences in overall accuracy on the tasks between the set size conditions.

**Table 1:** **Mean accuracy rates and response times by trial type in Experiment 1**.

**Set size**	***N***	**Age in years**	**Female**	**Accuracy (%)**				**Response times (ms)**			
				**Pure blocks**	**Non-switch**	**Switch**	**Switch cost**	**Pure blocks**	**Non-switch**	**Switch**	**Switch cost**
**YOUNGEST CHILDREN**											
Small	25	5;2 (0;8)	18	96.3 (5.9)	89.5 (13.2)	78.4 (16.9)	11.1 (12.3)	1482 (440)	1578 (365)	1916 (715)	337 (584)
Large	24	5;3 (0;9)	9	96.9 (4.1)	89.6 (11.0)	84.1 (12.8)	5.5 (12.1)	1362 (287)	1714 (382)	1773 (390)	59 (203)
**MIDDLE CHILDREN**											
Small	23	7;4 (0;7)	11	96.0 (5.5)	93.5 (7.9)	85.2 (7.1)	8.3 (10.2)	1104 (300)	1391 (391)	1493 (490)	102 (183)
Large	27	7;5 (0;6)	13	96.3 (4.8)	93.1 (8.6)	86.3 (12.2)	6.8 (10.6)	1186 (293)	1484 (364)	1571 (434)	87 (235)
**OLDEST CHILDREN**											
Small	22	9;9 (1;1)	12	97.0 (6.1)	94.6 (5.9)	91.2 (13.1)	3.4 (14.5)	914 (192)	1168 (318)	1258 (450)	91 (219)
Large	28	9;8 (1;0)	17	96.4 (5.8)	96.4 (5.2)	92.0 (7.2)	4.4 (8.2)	999 (293)	1239 (257)	1272 (310)	33 (180)

*For ease of reference, switch costs are also shown. Standard deviations are presented in parentheses*.

The analysis of RT data revealed a main effect of age, *F*_(2, 143)_ = 24.36, *p* < 0.001, η^2^ = 0.25. Bonferroni-adjusted *post-hoc* tests revealed that the youngest group was slower than both the middle and oldest age groups (*p*s < 0.01). The middle age group was also slower than the oldest age group (*p* < 0.01). Analysis of trial type revealed a significant switch cost, *F*_(1, 143)_ = 22.57, *p* < 0.001, η^2^ = 0.14, with slower performance on switch trials than on non-switch trials. RT switch costs were not significantly different between age groups (see Table 1 for a summary of the means).

**Figure 2:**
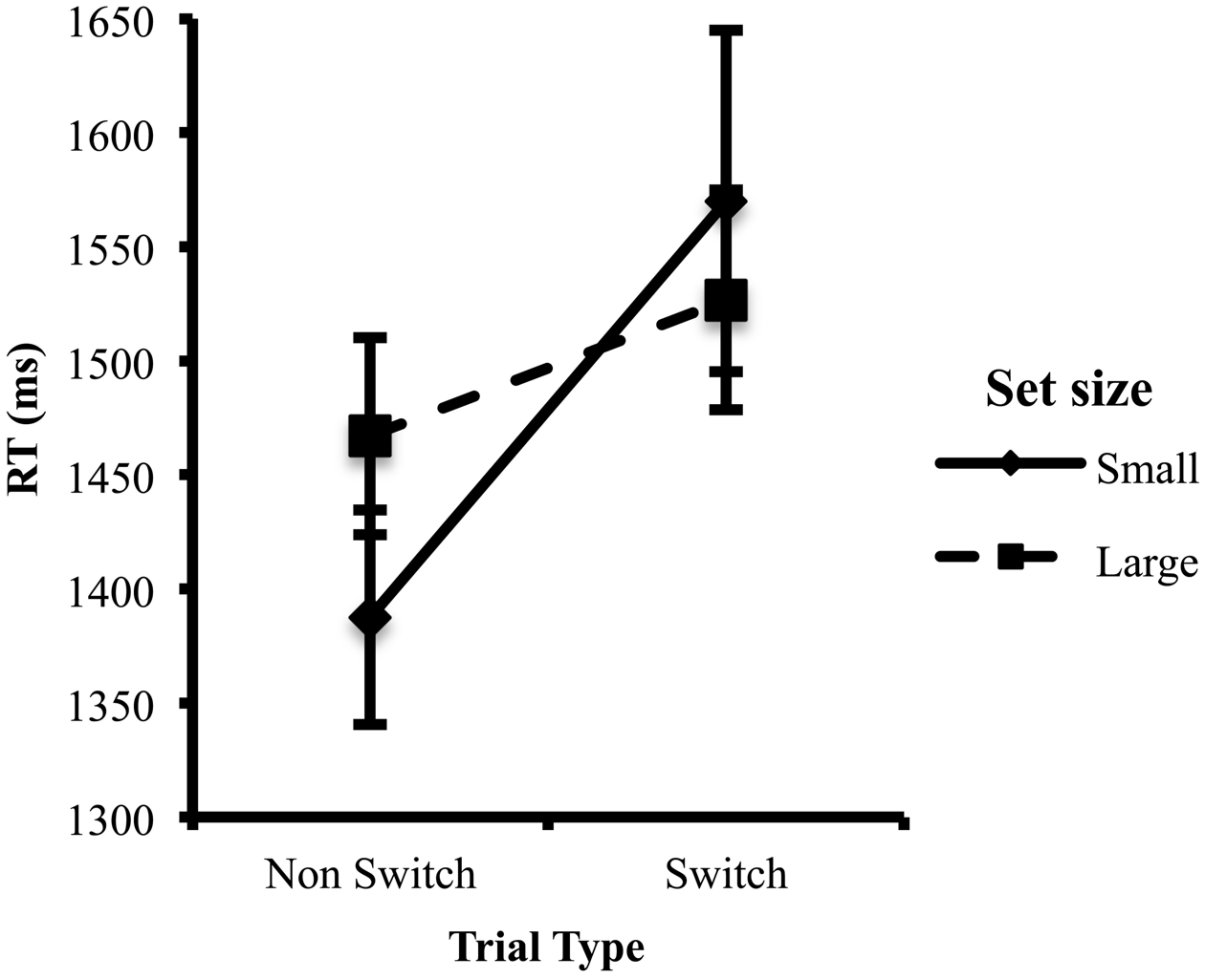
**Mean RT in the switch and non-switch trials as a function of the set size**. Error bars indicate the standard error of the mean.

There was an interaction between set size and trial type, *F*_(1, 143)_ = 5.84, *p* < 0.05, η^2^ = 0.04, such that switch costs were greater in the small set size condition than in the large set size condition (see Figure 2). To investigate whether set size affected response times on switch trials, on non-switch trials, or on both, separate Bonferroni-adjusted *post-hoc* tests were performed for RTs on Switch trials and non-switch trials, comparing the small and large set-size conditions. Descriptively, non-switch trials were faster in the small set size condition than the large set size condition (see Figure 2). Conversely, switch trials were slower in the small set size condition than the large set size condition. However, these differences were not significant (*p*s > 0.1). There was no overall effect of set size on RT, which indicates that the set size conditions did not differ in terms of processing speed.

Paired samples *t*-tests were conducted to determine whether RTs on switch and non-switch trials were significantly different for each set size condition (i.e., whether there was a significant switch cost). In the small set size condition, switch trials were significantly slower than non-switch trials, *t*_(69)_ = 4.57, *p* < 0.01. In the large set size condition, switch trials were marginally slower than non-switch trials, *t*_(78)_ = 1.91, *p* = 0.060.

To investigate whether there were more gradual age-related changes in cognitive flexibility in our sample, accuracy and RT switch costs (difference scores between switch and non-switch trials) were entered into a bivariate correlation analysis with age (mean-centered). Both types of switch cost were negatively correlated with age [accuracy: *r*_(147)_ = −0.18, *p* = 0.033; RT: *r*_(147)_ = −0.17, *p* = 0.037].

### Discussion

In Experiment 1, a switching paradigm with minimal incidental demands was used to investigate the effects of set size on cognitive flexibility during the early school years. The results showed significant age-related changes in accuracy and RT on both non-switch and switch trials. However, contrary to our predictions there were no significant differences in switch costs between the age groups. This is consistent with other studies that have found little or no change in switch costs over the early school period (Dibbets and Jolles, 2006; Karbach and Kray, 2007; Kray et al., 2012; though see also Chevalier and Blaye, 2009; Cragg and Nation, 2009). However, correlational analyses did reveal a developmental trend of reduced switch costs with age for both the accuracy and the RT data. This suggests that there are developmental changes to switch costs, but that these are gradual and may be harder to detect when data are categorized for analysis by age group. This gradual trend may explain the inconsistency in the literature with regards to age-related changes in switch costs.

In line with our predictions, when the set size was small, response-time switch costs were greater than when the set size was large. This indicates that a smaller set size leads to more interference between tasks, and is consistent with Kray et al.'s (2012) findings. It is also consistent with findings that show switch costs to be greater when there is more stimulus overlap between tasks (Waszak et al., 2003; Koch and Allport, 2006). These data clearly demonstrate, then, that a set size effect is apparent in children's cognitive flexibility. Furthermore, the difference between switch and non-switch trials was only marginally significant when the set size was large. This shows that the cognitive processes that contribute to switch costs are affected by the set size. Thus, to understand the processes that contribute to switch costs in children, it is necessary to understand what drives the set-size effect found in Experiment 1. The mechanisms that underpin this effect remain to be elucidated.

Experiment 1 identified that increasing the set size of the tasks reduced the cost of switching between them. We have identified two candidate cognitive mechanisms that could explain why increasing the set size reduces switch costs. The first explanation, which we refer to as the Rule Representation account, posits a mechanism in which set size affects the way that task rules are represented, which influences cognitive flexibility (Kharitonova et al., 2009). The larger set size may engender abstract, dimension-level representations of the task rules, whereas the smaller set size may engender stimulus-specific representations of the task rules. More abstract representations should lead to more flexible switching between tasks (Kharitonova et al., 2009).

The second explanation, which we refer to as the Stimulus-Task Priming account, posits a mechanism in which set size affects bottom-up priming of stimulus-to-task mappings (Waszak et al., 2003; Koch and Allport, 2006). This is because when the set size is small, individual stimuli repeat more often, both within and between the two tasks. This would be expected to lead to associations between stimuli and tasks which would both facilitate repeating a task from one trial to the next, and impair switching between tasks. In contrast, when the set size is large, individual stimuli repeat less often. This would be expected to lead to much less pronounced priming effects relative to a smaller set size. The direction of the means in Experiment 1 are consistent with this account insofar as non-switch trials were slower when the set size was large than when it was small, and switch trials were faster when the set size was large than when it was small. In adults, associations between stimuli and tasks can lead to switch costs even after 100 intervening trials (Waszak et al., 2003). The duration of stimulus-task associations in children is not yet known. The effect may last as long as it does in adults, or it may be limited to consecutive trials. To maximize the chances of detecting a stimulus-task priming effect—if it exists—the set size conditions differed both in terms of frequency of stimulus repetitions, and in terms of the number of intervening trials between repetitions. The small set size condition had many trials where stimuli repeated from one trial to the next. The large set size condition had no trials where stimuli repeated on consecutive trials. However, within each condition, the number of intervening trials was not systematically varied, so the duration of stimulus-task priming was not investigated here.

To test the Rule Representation account and the Stimulus-Task Priming account directly, it is necessary to tease apart two things: the rule representation effects that are initiated during the rule-learning phase, and the priming effects that occur during the task-switching phase. To do this, set size was varied independently in the rule-learning phase and in the task-switching phase. Set size was either large or small. This two-by-two design yielded four conditions, differing firstly according to the set size in the rule-learning phase, and secondly according to the set size in the later task-switching phase. Note that the Rule Representation and Stimulus-Task Priming accounts are not mutually exclusive. The set size effect observed in Experiment 1 may be best explained by one process, or by the other, or by both together. Experiment 2 seeks to address this question.

The Rule Representation account predicts that the set size in the rule-learning phase would have an effect on switch costs. Specifically, if the set size was large in the rule-learning phase, then children would be more likely to form more flexible dimension-level rule representations, and if the set size was small in the rule learning phase then children would be more likely to form less flexible stimulus-specific rule representations. Thus, a large set size during the rule-learning phase should lead to smaller switch costs than a small set size during the rule-learning phase. The Stimulus-Task Priming account predicts that the set size in the task-switching phase would have an effect on switch costs. Specifically, when the set size was small in the task-switching phase, then there would be more stimulus repetition between tasks than when the set size was large in the task-switching phase. This would lead to larger stimulus-task priming effects when the set size was small in the task-switching phase than when the set size was large in the task-switching phase. Thus, a large set size during the task-switching phase should lead to smaller switch costs than a small set size during the task-switching phase. Note that children form representations of the task rules quickly. Even at 3 years of age, children are capable of forming abstract representations of task rules after as few as six trials (Kharitonova et al., 2009).

Experiment 2 also explores developmental changes in the roles of rule representation and stimulus-task priming on cognitive flexibility with development. With age, children become more likely to spontaneously use abstract rule representations (Kharitonova et al., 2009; Kharitonova and Munakata, 2011). It was thus expected that having a large set size in the rule-learning phase would have the greatest facilitative effect on the youngest children in the sample, since they would be the least likely to spontaneously form abstract rule representations for the small set size condition, and thus the most likely to benefit from a condition that engenders it. Similarly, stimulus-task associations have been shown to be more robust in children than in adults (Hommel et al., 2011). If the strength of these associations follows a linear relationship through development, the effects of set size during the task-switching phase should also be strongest for the youngest children in our sample.

## Experiment 2

### Methods

#### Participants

Two hundred and forty three 5- to 10-year-old children (128 female) from two suburban primary schools in the UK took part: 84 in the youngest age group (5;3- to 6; 6-year-olds, *M* = 6;0, *SD* = 0;4, 46 females), 79 in the middle age group (7;2- to 8;6-year-olds, *M* = 8;0, *SD* = 0;4, 39 females) and 80 in the oldest age group (9;5- to 10;6-year-olds, *M* = 10;0, *SD* = 0;4, 42 females). Six further children were excluded either because they failed to follow instructions (*N* = 3) or because of missing data (*N* = 3). Participants had no known developmental disorders or special educational needs. Participants were randomly assigned to one of the four conditions. All the children were tested individually in a quiet room in their schools. Parental consent for participation in this research was obtained for all participants. The experimental procedure was approved by the Department of Psychology Ethics Committee at the University of Sheffield.

#### Materials and procedure

The stimuli and materials for Experiment 2 were the same as for Experiment 1, except that a Dell T7570 laptop running E-Prime v1.2 (Psychological Software Tools, Pittsburgh, PA) software was connected to an ATM-152ROHACB2D touch screen monitor. Set size was varied using a 2 × 2 design with two levels of set size (small and large) varying across the two phases of the experiment (the initial rule-learning phase, and the subsequent task-switching phase). This resulted in four conditions: the small:small condition had a small set size in the rule-learning phase and a small set size in the task-switching phase; the small:large condition had a small set size in the rule-learning phase and a large set size in the task-switching phase; the large:small condition had a large set size in the rule-learning phase and a small set size in the task-switching phase; and the large:large condition had a large set size in the rule-learning phase and a large set size in the task-switching phase. (Note that the small:small condition and the large:large condition were identical to the small and large set size conditions in Experiment 1.) Stimulus selection was constrained in the same way as in Experiment 1.

### Results

The mean accuracy and mean log-transformed response time (RT) were calculated for each child for each trial type. Trials with RT less than 200 ms or greater than 10,000 ms, and trials 2.5 standard deviations or greater away from the individual's mean RT for that type of trial (5.0%) were excluded from the response-time analysis. Response-time analyses included only correct trials that followed a correct trial. The mean number of trials entered into the analysis did not differ between the set size conditions, nor between switch and non-switch trials. Younger children contributed fewer trials to the analysis than older children because of higher error rates (*M*s = 23.1, 25.5, and 24.3 trials entered for the youngest, middle and oldest age groups respectively).

All analyses were performed separately for each dependent variable of interest: mean accuracy and mean log-transformed RTs. To assess switch costs, a mixed measures ANOVA was performed with trial type (non-switch vs. switch) as a within-participants factor, and age (youngest vs. middle vs. oldest), set size in the rule-learning phase (small vs. large) and set size in the task-switching phase (small vs. large) as between-participant factors.

The analysis of accuracy data revealed a main effect of age, *F*_(2, 231)_ = 15.72, *p* < 0.001, η^2^ = 0.0.12. Bonferroni-adjusted *post-hoc* tests showed that the youngest children were less accurate than both the middle children and the oldest children (*p*s < 0.001; see Table 2 for a summary of the means). There was no significant difference between the accuracy of the middle and oldest children. Analysis of trial type revealed a significant switch cost, *F*_(1, 231)_ = 75.23, *p* < 0.001, η^2^ = 0.24, with less accurate performance on switch trials than non-switch trials. There was an interaction between age group and trial type, *F*_(2, 231)_ = 3.41, *p* < 0.05, η^2^ = 0.03. Bonferroni-adjusted *post-hoc* tests showed that the youngest children had greater switch costs for accuracy than the oldest children (*p* < 0.05). No other age comparisons were significant. There was also a three-way interaction between age, trial type and set size in the rule-learning phase, *F*_(2, 231)_ = 3.15, *p* < 0.05, η^2^ = 0.03. Bonferroni-adjusted *post-hoc* tests showed that for the youngest age group, switch costs were greater when the set size in the rule-learning phase was large than when it was small (*p* < 0.05). Set size in the rule-learning phase did not affect switch costs for the middle or oldest age groups (*p*s > 0.1). There was no effect of set size on the accuracy of performance. This indicates that there were no baseline differences in overall accuracy on the tasks between the set size conditions.

**Table 2:** **Mean accuracy rates and response times by trial type in Experiment 2**.

**Set size**	***N***	**Age in months**	**Female**	**Accuracy (%)**				**Response times (ms)**			
				**Pure blocks**	**Non-switch**	**Switch**	**Switch cost**	**Pure blocks**	**Non-switch**	**Switch**	**Switch cost**
**YOUNGEST CHILDREN**											
Small:Small	21	6;1 (0;4)	11	96.8 (5.6)	89.0 (14.9)	86.6 (17.7)	2.4 (12.3)	1339 (316)	1697 (427)	1785 (428)	88 (235)
Small:Large	19	6;1 (0;4)	12	93.8 (8.5)	89.8 (17.5)	84.2 (12.6)	5.6 (10.4)	1321 (389)	1679 (339)	1772 (458)	93 (306)
Large:Small	23	5;11 (0;4)	13	98.6 (4.1)	92.9 (7.6)	82.6 (14.6)	10.3 (11.1)	1540 (340)	1757 (424)	1871 (518)	115 (304)
Large:Large	21	6;0 (0;4)	10	95.2 (6.8)	96.4 (7.0)	88.5 (12.7)	7.9 (10.8)	1410 (387)	1761 (503)	1700 (387)	−62 (230)
**MIDDLE CHILDREN**											
Small:Small	20	7;11 (0;3)	9	98.3 (3.4)	98.4 (2.8)	93.8 (6.7)	4.6 (6.2)	1167 (385)	1312 (301)	1389 (345)	77 (215)
Small:Large	20	7;11 (0;4)	9	98.3 (4.4)	98.1 (4.1)	91.5 (9.0)	6.7 (8.4)	1170 (280)	1427 (425)	1529 (376)	102 (248)
Large:Small	20	7;11 (0;4)	11	97.9 (4.6)	96.3 (5.1)	93.2 (5.8)	3.0 (7.4)	1404 (556)	1355 (367)	1499 (342)	144 (173)
Large:Large	19	8;0 (0;4)	10	96.7 (6.8)	95.4 (5.8)	90.4 (9.2)	5.0 (9.7)	1261 (257)	1403 (321)	1426 (271)	23 (233)
**OLDEST CHILDREN**											
Small:Small	19	10;0 (0;3)	10	98.2 (4.5)	98.4 (4.6)	95.7 (7.6)	2.7 (4.9)	932 (162)	1140 (243)	1208 (223)	69 (164)
Small:Large	20	10;0 (0;3)	10	99.2 (3.7)	95.6 (5.0)	93.2 (6.7)	2.4 (4.5)	934 (171)	1184 (253)	1252 (243)	68 (145)
Large:Small	20	10;0 (0;4)	11	99.2 (2.6)	95.9 (5.1)	92.4 (6.1)	3.6 (6.5)	1076 (280)	1186 (401)	1293 (314)	107 (239)
Large:Large	21	10;0 (0;3)	11	97.6 (4.7)	97.6 (3.1)	94.1 (6.7)	3.5 (6.3)	1006 (139)	1213 (243)	1228 (207)	15 (196)

*For ease of reference, switch costs are also shown. Standard deviations are presented in parentheses*.

The analysis of RT data revealed a main effect of age, *F*_(2, 231)_ = 53.78, *p* < 0.001, η^2^ = 0.32. Bonferroni-adjusted *post-hoc* tests revealed that the youngest group was slower than both the middle and oldest age groups (*p*s < 0.01). The middle age group was also slower than the oldest age group (*p* < 0.01). Analysis of trial type revealed a significant switch cost, *F*_(1, 231)_ = 33.84, *p* < 0.001, η^2^ = 0.13, with slower performance on switch trials than non-switch trials (see Table 2 for the mean transformed RTs).

**Figure 3:**
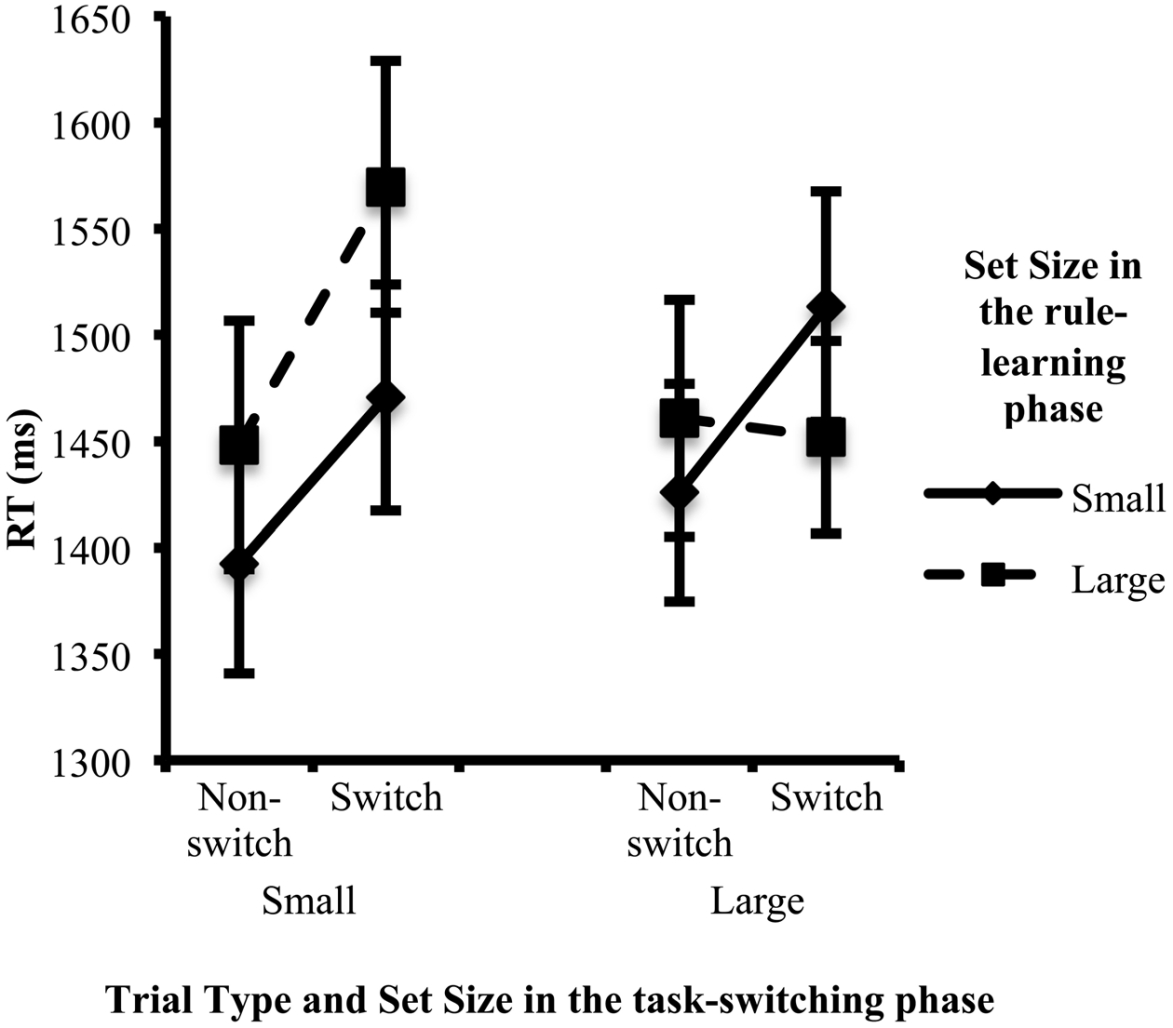
**Mean RT in the switch and non-switch trials as a function of the set size in the rule-learning and task-switching phases**. Error bars indicate the standard error of the mean.

Two further significant interactions were revealed in the RT analysis. First, the set size in the task-switching phase interacted with the trial type *F*_(1, 231)_ = 7.03, *p* < 0.01, η^2^ = 0.03. Overall, switch costs were larger when the set size in the task-switching phase was small (*M* = 100 ms) than when the set size in the task-switching phase was large (*M* = 39 ms). Separate Bonferroni-adjusted *post-hoc* tests were performed for RTs on Switch and Non-switch trials comparing conditions with a small set size in the task-switching phase and those with a large set size in the task-switching phase. Descriptively, when there was a small set size in the task-switching phase, non-switch trials were faster than when there was a large set size in the task-switching phase. Conversely, when there was a small set size in the task-switching phase, switch trials were slower than when there was a large set size in the task-switching phase (see Figure 3). However, these differences were not significant (*p*s > 0.1).

Second, there was a three-way interaction between the set size in the rule-learning phase, the set size in the task-switching phase and the trial type, *F*_(1, 231)_ = 7.73, *p* < 0.01, η^2^ = 0.03. Two pairwise Bonferroni-adjusted *post-hoc* tests were conducted. One compared switching costs between the two conditions with a large set size in the rule-learning phase (the large:small and large:large conditions). The other compared the two conditions with a small set size in the rule-learning phase (the small:small and small:large conditions). When the set size in the rule-learning phase was large, switch costs were affected by the set size in the task-switching phase (*p* < 0.05): switch costs were larger in the large:small condition (*M* = 122 ms) than in the large:large condition (*M* = −9 ms; this value is negative because switch trials were faster than non-switch trials in this condition). When the set size in the rule-learning phase was small, switch costs were not affected by the set size in the task-switching phase (*p* > 0.1, see Figure 3). There was no overall effect of set size on RT, which indicates that the groups did not differ in terms of processing speed.

Paired samples *t-*tests were conducted for each set size condition to determine whether RTs on switch and non-switch trials were significantly different (i.e., whether there was a significant switch cost). For the small:small, small:large and large:small conditions, switch trials were slower than non-switch trials, *t*s > 1, *p*s < 0.01. For the large:large condition, RTs on switch and non-switch trials did not differ significantly.

To test whether the set-size effect found in Experiment 1 was replicated in Experiment 2, planned comparisons of switch costs in the small:small and large:large conditions were conducted. These showed that there were greater switch costs in the small:small condition than in the large:large condition, *t*_(109)_ = 2.30, *p* < 0.05. Thus, the set-size effect reported in Experiment 1 was also replicated in Experiment 2.

Finally, to investigate whether there were more gradual age-related changes in cognitive flexibility in our sample, accuracy and RT switch costs (difference scores between switch and non-switch trials) were entered into a bivariate correlation analysis with age. Accuracy switch costs were negatively correlated with age (mean-centered), *r*_(241)_ = −0.16, *p* = 0.011. However, reaction time switch costs were not related to age: *r*_(241)_ = −0.056, *p* = 0.38.

### Discussion

Experiment 2 replicated the key finding of Experiment 1, namely that with school-aged children, a small set size leads to larger switch costs than a large set size. In addition, Experiment 2 extended our understanding of how this effect comes about by testing the two accounts of this effect: the Rule Representation account and the Stimulus-Task Priming account. There was no support for the Rule Representation account: a large set size in the rule-learning phase did not lead to reduced switch costs compared to a small set size. Conversely, there was support for the Stimulus-Task Priming account: a large set size in the task-switching phase led to reduced switch costs compared to a small set size in the task-switching phase. However, the presence of a three-way interaction between trial type, set size in the rule-learning phase and the set size in the task-switching phase suggests that there is a more complex story to be told. Further discussion of these accounts in light of the current findings can be found in the general discussion.

Contrary to previous research, Experiment 2 did not provide evidence for age-related changes in stimulus-task priming. Previous research has shown that 9- and 10-year-old children are more susceptible to stimulus-task priming than young adults (Hommel et al., 2011). Experiment 2 included children up to 10 years of age. The lack of interaction between age, trial type and the set size in the task-switching phase suggests that developmental changes in the ability to overcome stimulus task priming may be limited to later childhood and adolescence. An alternative explanation is that stimulus-task priming may be developmentally invariant. This view is consistent with research demonstrating that implicit learning processes such as priming of stimulus-response associations does not change from infancy to adulthood (Amso and Davidow, 2012).

## General discussion

The two experiments presented in this paper shed important new light on how set size influences cognitive flexibility during development. They extend previous research by showing that children's switch costs can be reduced by increasing the set size. This finding is directly relevant to preschool research into cognitive flexibility. The most widely used paradigm, the DCCS (Zelazo et al., 1996; Müller et al., 2006), typically uses a small set size (usually comprising four stimuli in total). Experiment 1 and 2 indicate that using a small set size is likely to be particularly difficult for children, and that studies using such paradigms may systematically underestimate children's cognitive flexibility.

The present findings build on the work of Kray et al. (2012) who showed that when children switched between tasks, they experienced less interference when there was a large set size than when there was a small set size. The two experiments presented in this paper extend those findings to show that during the early school years, switch costs can also be reduced when the set size is increased. By manipulating set size in a paradigm that has minimal incidental demands and which uses simple perceptual categorization tasks, these experiments showed a robust effect of set size on switch costs for children from 4 to 12 years.

The Rule Representation account of the set size effect was not supported. According to this account, the set size affects the way task rules are represented which affects switch costs. A small set size during the rule-learning phase was expected to result in less flexible stimulus-specific rule representations while a large set size during the rule-learning phase was expected to result in more flexible dimension-level rule representation. None of the predictions derived from the Rule Representation account were borne out in the findings of Experiment 2. Indeed, directly contrary to the prediction of the Rule Representation account, the youngest children's accuracy switch costs were greater when there was a large set size in the rule-learning phase than when there was a small set size in the rule-learning phase. These findings suggest that the way that task rules are represented does not drive the facilitative effects of a large set size on switch costs.

Furthermore, the lack of effect of set size in the rule-learning phase on switch costs raises questions over the robustness of the association between abstraction and flexibility. Previous research has shown that children who form dimension-level representations of task rules have better cognitive flexibility than children with stimulus-specific rule representations (Kharitonova et al., 2009; Kharitonova and Munakata, 2011). However, in Experiment 2, there was no main effect of set size in the rule-learning phase. Engendering dimension-level rule representation by presenting participants with a large set size in the rule-learning phase did not increase later flexibility during the task-switching phase. It is possible that the rule-learning phase was too short to engender stable rule representations that persisted into the task-switching phase. However, research with children as young as 3 years demonstrates that dimension level and stimulus-specific rule representations can be formed after six trials (Kharitonova et al., 2009). In the two experiments presented in this paper, even the youngest children made very few mistakes during the rule-learning phase. This suggests that the rules were intuitive and easy to learn. It remains a question for future research whether more trials during the rule-learning phase would lead to more persistent representations of task rules.

The Stimulus-Task Priming account is supported by the findings of the two experiments presented in this paper. According to this account, the set size in the task-switching phase affects the amount of stimulus-task priming that occurs which affects switch costs. More stimulus repetition occurs when the set size was small in the task-switching phase than when the set size was large in the task-switching phase. This was expected to result in more stimulus-task priming and so greater switch costs when the set size was small in the task-switching phase than when the set size was large in the task-switching phase. First, in both experiments, when the set size in the task-switching phase was small, non-switch trials were faster and switch trials were slower than when the set size in the task-switching phase was large (although these differences were not statistically significant). This was consistent with predictions from the Stimulus-Task Priming account, since stimulus-task priming should be facilitative for non-switch trials and detrimental for switch trials (Waszak and Hommel, 2007). Second, in Experiment 2 there was a main effect of set size in the task-switching phase. This provides evidence that the link between set size and cognitive flexibility is mediated by stimulus-task priming that occurs as a result of stimulus repetition during the task-switching phase. This bottom-up process includes priming as a result of stimulus repetition both within task (which facilitates task repetition) and between the two tasks (which impairs task switching). The findings of this study suggest that young children's cognitive flexibility is affected by stimulus-task priming and that this priming contributes to switch costs.

However, two findings from Experiment 2 suggest that switch costs cannot be solely driven by bottom-up processes. First, switch costs were found in the near-absence of stimulus-task priming, which suggests that top-down processes may also contribute to switch costs. Specifically, in Experiment 2, significant switch costs were found in the small:large condition. Recall that in this condition, very little stimulus repetition occurs during the task-switching phase. The switch costs that occur here are thus unlikely to be driven by stimulus-task priming. Second, there was a three-way interaction between trial type, set size in the rule-learning phase and set size in the task-switching phase. This shows that set size during the rule-learning phase has carryover effects that moderate the effects of set size in the task-switching phase on switch costs.

Both of the above findings can be explained by the same top-down mechanism. We suggest that exposure to a small set size in the rule-learning phase led children to expect high levels of conflict between the tasks in the task-switching phase. This would have promoted more engagement of top-down control processes. It is plausible that children will prepare more for task switches under conditions where they expect high levels of conflict. Thus, switch costs likely occur in the small:large condition as a result of greater engagement from top-down control processes on switch trials than non-switch trials. These same top-down control processes likely attenuate the effects of set size in the task switching phase. This explains the three-way interaction found in the RT analysis. This explanation is consistent with the findings of Kray et al. (2012), who found that children adapted better to conflict with a small set size than a large set size.

Together, these findings suggest that a combination of top-down and bottom-up processes contribute to switching costs in early school age children, and that these are differentially affected by manipulations of set size. This proposal is entirely consistent with findings from the adult literature which suggest that stimulus-task priming only partially accounts for the cost of switching from one task to another (for a review, see Vandierendonck et al., 2010). However, the developmental trajectory of this interaction is still uncertain. Although we expected to find a shift from bottom-up to top-down processing with development (Munakata et al., 2012), our findings did not wholly bear this out. We found little evidence for reliable developmental change in cognitive flexibility. It is true that accuracy switch costs were negatively correlated with age in both experiments. However, RT switch costs were negatively correlated with age in Experiment 1, but not related to age in Experiment 2. This inconsistency may in part have been due to the variability of performance in our youngest age group (see standard deviations in Tables 1, 2). It is common for response times to be variable for children of this age (for example see Chevalier and Blaye, 2009). This variability may be exacerbated by heterogeneity in cognitive strategies employed by 5- and 6-year-olds when engaged in task switching (Dauvier et al., 2012).

Clearly, set size influences different facets of cognitive flexibility in conflicting ways. Having a smaller set size in the task-switching phase impaired cognitive flexibility (leading to larger RT switch costs) by increasing the amount of stimulus-task priming that subjects were subjected to. Conversely, having a smaller set size in the rule-learning phase directly enhanced cognitive flexibility for the youngest children (leading to smaller accuracy switch costs), and attenuated the effects of stimulus-task priming on response time switch costs by increasing engagement of top-down control processes. This highlights the complexity of cognitive flexibility, demonstrating that a multitude of processes must work in harmony to produce flexible behavior (Cragg and Nation, 2010; Ionescu, 2012). However, it also raises the interesting possibility that there are multiple routes by which cognitive flexibility can be influenced, and that set size may act in contrasting ways depending on the stage in the task.

Such an observation is particularly relevant for those involved in educational research. There are many situations when cognitive flexibility is necessary for academic performance (Bull and Scerif, 2001; Blair and Razza, 2007; Bull et al., 2008). The current paper suggests two distinct ways to promote cognitive flexibility that can be applied to educational settings. First, by reducing the amount of stimulus repetition, it is possible to reduce the amount of interference between tasks (Kray et al., 2012) and to reduce the costs of switching from one task to another. For example, it may be possible to improve children's ability to switch from addition to subtraction problems when teaching arithmetic by ensuring that a large set of numbers are used for the two sets of problems. Second, it may also be possible use a small set size to promote children's top-down control by highlighting the conflict between tasks. Our findings suggest that for school-age children this is most effective if done during situations with relatively low control demands, such as during the rule-learning phase in our paradigm. This will enable children to overcome some of the effects of stimulus-task priming when interference between tasks is high.

### Future directions

A number of questions remain regarding the relationship between set size and stimulus-task priming. First, stimulus-task priming may occur at the perceptual or conceptual level (Waszak et al., 2004). The manipulation of set size in the experiments presented here changed both the perceptual and conceptual set size concurrently, so the relative roles of conceptual and perceptual priming cannot be distinguished. Future research may address this question by using tasks with a large perceptual set but a small conceptual set (for example, many different shades of red and blue stimuli).

Second, while it is now clear that bottom-up priming plays a central role in the set size effect, it remains to be determined what kind of priming is at work. There are at least two types of bottom-up priming that may be occurring. Stimulus repetition across tasks may cause the competing task to be positively primed (termed “competitor priming”) and the relevant task to be negatively primed (termed “negative priming” Hübner et al., 2003; Waszak et al., 2005). It is not yet known whether set size influences competitor priming or negative priming or both, nor whether these influences change with development.

Third, it is not known how stable the bindings between stimuli and tasks are. Stimulus-task priming may be subject to decay, so only driven by stimulus repetition from one trial to the next or within a short time-frame (Allport and Wylie, 2000). Alternatively, bindings may be stable and long-lived, so stimulus-task priming may be driven by stimulus repetition across the tasks, irrespective of intervening trials (Waszak et al., 2003). While research has begun to address many of these questions in adulthood, the nature of bottom-up influences on children's cognitive flexibility is still poorly understood. Due to the limited number of trials entered into the analysis, it was not possible to explore the stability of the stimulus-task bindings in the experiments presented in this paper. Future research should systematically manipulate the delay between pairings of stimuli and tasks to determine the longevity of stimulus-task priming effects.

The relationship between set size and top-down control processes is also likely to be a fruitful pathway for further research. The primary method for studying top-down control processes during task switching is through manipulation of the time allowed to prepare for each trial between the onset of the task cue and the task stimuli (Meiran, 1996; Cepeda et al., 2001). In the experiments reported in this paper, the task cue and the prompt stimulus were presented simultaneously, so participants were not able to prepare in advance for the task. This means that task preparation that occurred following the onset of the cue was likely to impact on response times. By manipulating both preparation time and set size, future research will be able to determine unequivocally whether having a smaller set size during the rule-learning phase increased children's engagement of top-down control processes.

## Conclusion

The two experiments presented in this paper demonstrate that set size influences cognitive flexibility during childhood. When tasks have a large set size, the costs of switching are smaller than when tasks have a small set size. This is of practical importance because it suggests that children's cognitive flexibility may have been underestimated relative to adults', since paradigms used with children typically have a smaller set size than those used with adults. Furthermore, Experiment 2 furthered theoretical understanding of cognitive flexibility during development by elucidating the cognitive mechanisms that drive this effect. First, it was shown that differences in the amount of stimulus-task priming that occurs drive the set size effect found in Experiment 1. Second, it was shown that set size also influences children's engagement in top-down control processes. This engagement had a direct effect on the youngest children's cognitive flexibility, and attenuated the effects of stimulus-task priming in across the sample. Further exploration of both bottom-up and top-down effects of set size on cognitive flexibility will continue to elucidate the factors that contribute to the costs of task switching in children.

## Author contributions

Lucy Cragg designed Experiment 1. Lily FitzGibbon and Daniel J. Carroll designed Experiment 2. Data collection was carried out by Lucy Cragg and Lily FitzGibbon for Experiments 1 and 2 respectively. Lily FitzGibbon carried out data analysis of Experiments 1 and 2. Lily FitzGibbon drafted the manuscript and Lucy Cragg and Daniel J. Carroll provided critical revisions. Lily FitzGibbon, Lucy Cragg, and Daniel J. Carroll have all approved the final version of the manuscript and agree to be accountable for all aspects of the work.

### Conflict of interest statement

The authors declare that the research was conducted in the absence of any commercial or financial relationships that could be construed as a potential conflict of interest.
